# The Sirtuin 3 Expression Profile Is Associated with Pathological and Clinical Outcomes in Colon Cancer Patients

**DOI:** 10.1155/2014/871263

**Published:** 2014-07-01

**Authors:** Chunyuan Liu, Zonghai Huang, Hong Jiang, Fujun Shi

**Affiliations:** ^1^Department of General Surgery, Zhujiang Hospital, Southern Medical University, Guangzhou, Guangdong 510282, China; ^2^Department of General Surgery, Affiliated Hospital of Binzhou Medical University, Binzhou, Shandong 256600, China

## Abstract

*Aim.* To investigate the correlation between Sirtuin 3 (SIRT3) expression and the clinical outcome of patients with colon cancer. *Methods*. The tumor specimens from 127 patients with colon cancer were obtained for SIRT3 immunohistochemical staining. Patients were followed up. In *in vitro* study, SIRT3 gene was inhibited to observe the effects of SIRT3 on the biological behavior of cultured colon cells. *Results*. The SIRT3 expression level was found to be significantly associated with the lymph node metastasis (*P* < 0.001) and tumor stages (*P* < 0.001). The colon cancer-specific survival was 64.6% among patients with high SIRT3 expressions and 88.6% among patients with low SIRT3 expressions (log-rank *P* = 0.016). The overall survival was 80.2% among patients with low SIRT3 expressions and 55.9% among patients with high SIRT3 expressions (log-rank *P* = 0.002). *In vitro* study showed that silencing of SIRT3 gene inhibited the proliferation, invasion, and cells migration but increased the apoptosis in the cultured colon cell lines. *Conclusion*. This study provides evidence supporting that SIRT3 is closely associated with the clinical outcomes of colon cancer. SIRT3 may be considered as a marker for colon cancer.

## 1. Introduction

Colon cancer is a worldwide health problem with 655,000 deaths per year [1, 2]. In China, colon cancer remains a major cause of cancer-related death [3, 4]. Despite the development of new treatment in recent decades, the five-year survival rate of advanced colon cancer patients remains very poor because of insensitivity to chemotherapy and more easily prone to recurrence [5–7]. Although a number of biological markers have been reported to be related to colon cancer [8–10], exploiting new markers to predict the clinical features and outcome of colon cancer is still in need.

Sirtuin 3 (SIRT3) is a member of Sirtuin family, the mammalian homologues of the silent information regulator 2, first discovered in* Saccharomyces cerevisiae* as a nicotinamide adenine dinucleotide- (NAD-) dependent histone deacetylase [11, 12]. Recently the association between SIRT3 and tumorigenesis has raised wide attention. On one hand, some studies revealed that SIRT3 is a tumor promoter that prompts tumorigenesis. For example, SIRT3 prevents growth arrest and senescence in bladder carcinoma cells by abrogating p53 activity [13]. In oral squamous cell carcinoma (OSCC) cell lines, downregulation of SIRT3 inhibited OSCC cell growth and proliferation and increased OSCC cell sensitivity to radiation and cisplatin treatments* in vitro* [14]. Studies have also shown that SIRT3 downregulation reduced tumor burden* in vivo* [14]. The overexpression of SIRT3 has been found in lymph node-positive breast cancer [15]. On the other hand, some studies showed that SIRT3 may be a tumor suppressor [14]. SIRT3 inhibits hepatocellular carcinoma cell growth through reducing Mdm2-mediated p53 degradation [16]. SIRT3 serves as a mitochondria-localized tumor suppressor and is required for integrity and metabolism of mitochondria during stress [17]. Thus, the function of SIRT3 varies in normal and tumor tissues and may be cell- and tumor-type specific.

Surprisingly, the correlation of SIRT3 expression and human colon cancer remains unknown. In this study, we detected the SIRT3 expression in tumor samples from colon cancer patients and analyzed its correlation to clinical outcomes in those patients. In addition, we performed the* in vitro* study to unfold the effects of SIRT3 on the biological behaviors, including proliferation, apoptosis, migration, and tumor invasion, of cultured colon cell lines.

## 2. Methods

### 2.1. Patient Samples and Followup

Tissue samples were collected from patients with colon cancer who underwent operation in the Department of General Surgery at the Affiliated Hospital of Binzhou Medical University from July 2005 to December 2010. Formalin-fixed and paraffin-embedded tissue specimens from 127 patients diagnosed with colon cancer were included. All the patients received fluorouracil-based adjuvant chemotherapy after surgical resection. Clinical and pathological classification and staging were determined according to the American Joint Committee on Cancer criteria [18]. Patients were observed until death or December 2012, whichever came first. Patients were followed up for the median of 35.3 months (ranged, 3.5–58.8 months). The study protocol was approved by the ethics committee of our hospital. Written informed consent was obtained from each participant before data collection.

### 2.2. Immunohistochemical Staining and Evaluation

The cancer tissues were fixed, paraffin-embedded, and cut to 5 *μ*m thick sections for immunohistochemistry. Briefly, the slides were incubated with SIRT3 and CD31 primary antibodies (both 1 : 200, Santa Cruz Biotechnology, Santa Cruz, CA). The immunoreactive products were visualized by the catalysis of 3,3′-diaminobenzidine [19]. SIRT3 protein expression was evaluated using the immunoreactive-score (IRS) system (combining positive cell ratio and staining intensity). An IRS score of ≥6 was used to define tumors as the high expression of SIRT3, and an IRS score of <6 was used to indicate the low SIRT3 expression [20].

### 2.3. Tumor Cell Line Culture and Small Interfering RNA Transfection (siRNA)

Two human colon carcinoma cell lines, SW480 and DLD-1 cells, were cultured in MEM, supplemented with 10% fetal bovine serum (FBS) and 1% antibiotic-antimycotic solution at 37°C in a humid incubator with 5% CO_2_. Nonspecific control siRNA or SIRT3 siRNA (sequence: CAT CCC TAC ATG CAG ATG AAA) was transfected for 48 hours using siLentFect Lipid Reagent (Bio-Rad, Hercules, CA, USA) according to the manufacturer's instructions.

#### 2.3.1. Quantitative Real-Time PCR (q-PCR) Analysis of SIRT3

Total RNA (1 *μ*g) was reversely transcribed by QuantiTect Reverse Transcription Kit (Qiagen, Hilden, Germany) from cells after siRNA transfection and cDNA was synthesized. Quantitative RT-PCR was carried out using the FastStart Universal SYBR Green Master (Roche) on an Applied Biosystems ABI 7900 Real-Time PCR System (Applied Biosystems, Foster City, CA, USA). The oligonucleotide primers for human SIRT3 and glyceraldehyde-3-phosphate dehydrogenase (GAPDH) were as follows: SIRT3-F 5′-ACCCAGTGGCATTCCAGAC-3′; SIRT3-R 5′-GGCTTGGGGTTGTGAAAGAAG-3′; GAPDH-F 5′-GAGTCAACGGATTTGGTCGT-3′; GAPDH-R 5′-GACAAGCTTCCCGTTCTCAG-3′. The gene expression level was normalized using GAPDH as an internal reference gene, and the average relative change was calculated using 3–5 determinations by relative quantification, applying the delta-delta cycle threshold method.

#### 2.3.2. Western Blot Analysis

The cancer receiving after siRNA transfection was lysed for western blot assay. After immunoblot analysis, membranes were immunoblotted with anti-SIRT3, antihypoxia-inducible factor 1 alpha (HIF1*α*), antivascular endothelial growth factor A (VEGF-A), and anti-GAPDH (all at 1:1000 dilution, Santa Cruz Biotechnology, Santa Cruz, CA). Membranes were then washed and incubated with a secondary antibody coupled to horseradish peroxidase.

### 2.4. Cell Proliferation Assay

Cellular proliferation was analyzed after siRNA transfection using a WST-8 Cell Counting Kit-8 (CCK-8) (Beyotime, Nantong, China). 3 × 10^3^ cells were seeded in 96-well plates and incubated for 24 h. 10 *μ*L CCK-8 solution was added to each well and the cultures were incubated at 37°C for 1 h. Absorbance at 450 nm was measured on an ELX-800 spectrometer reader (Bio-Tek Instruments, Winooski, USA). This assay was repeated 3 times.

### 2.5. Cell Migration Assay

After siRNA transfection, cell migration was determined by using a modified two-chamber migration assay with a pore size of 8 *μ*m. For the migration assay, 1 × 10^5^ cells were seeded on the upper compartment of 24-well Transwell culture chamber. After 24-hour incubation, nontraversed cells were removed from the upper surface of the filter carefully with a cotton swab. Traversed cells on the lower side of the filter were stained with crystal violet and then counted. This assay was repeated 3 times.

### 2.6. Cell Invasion Assay

The invasion assay was performed using a modified two-chamber plates. 30 *μ*L of 50 mg/mL Matrigel (BD Biosciences, Mississauga, Canada) in serum-free medium was added to the upper compartment of 24-well Transwell culture chamber. 1 × 10^5^ cells suspended in 200 *μ*L of serum-free medium were seeded on the upper compartment, and 600 *μ*L of complete medium was added to the lower compartment. After 24-hour incubation at 37°C, cells were fixed with methanol. Noninvaded cells were removed from the upper surface of the filter carefully with a cotton swab. Invaded cells on the lower side of the filter were stained with crystal violet and counted afterwards. This assay was repeated 3 times.

### 2.7. ROS Formation Assays in Cultured Cells

To detect the intracellular ROS production in cancer cells, 1 × 10^4^ cells were seeded in chamber wells and cultured for 24 hours to reach >80% confluence. Then CM-H2DCFDA (10 ìmol/L, Molecular Probes, USA) was added to chamber wells for 30 minutes. The nuclei were counterstained with 4,6-diamino-2-phenylindole (DAPI).

### 2.8. Terminal Deoxynucleotidyl Transferase dUTP Nick End Labeling (TUNEL) Assay

To induce apoptosis, the cultured cells received serum starvation for 48 hours. The in situ DeadEndTM Colorimetric Apoptosis Detection System (Promega, Madison, WI) was used according to the manufacturer's instructions. Sample slides were washed in PBS and then fixed in 4% paraformaldehyde solution. Sections were incubated with terminal deoxynucleotidyl transferase enzyme in a humidified chamber at 37°C for 60 min. The reaction was terminated by transferring the slides to 2× sodium citrate saline solution. The sections were counterstained with DAPI. For quantitative analysis, 5 fields per section were selected. Apoptosis was indexed by counting TUNEL positive cells per 100 nuclei per section.

### 2.9. Statistical Analysis

A two-sided Fisher exact test or *χ* ^2^ test was performed to analyze the potential association between SIRT3 expression and clinical parameters. The cumulative survival time was computed using the Kaplan-Meier method and compared by the log-rank test. Colon cancer-specific survival was defined from colon cancer diagnosis to time of death, which was related to colon cancer or censored on the last known alive date, or death due to other causes. Overall survival (OS) is defined as the time between study registration and a patient's death. Univariate and multivariate analyses were based on the Cox proportional hazards regression model. To evaluate the amount of protein expression, the Raytest TINA software (http://www.raytest.de/service/raytest_catalog.html) was used for the densitometric analysis in the western blot assay as described previously [21]. *P* < 0.05 was considered statistically significant. Analyses were performed using the statistical software package SAS 9.32 (SAS Inc., USA).

## 3. Results

**Figure 1:**
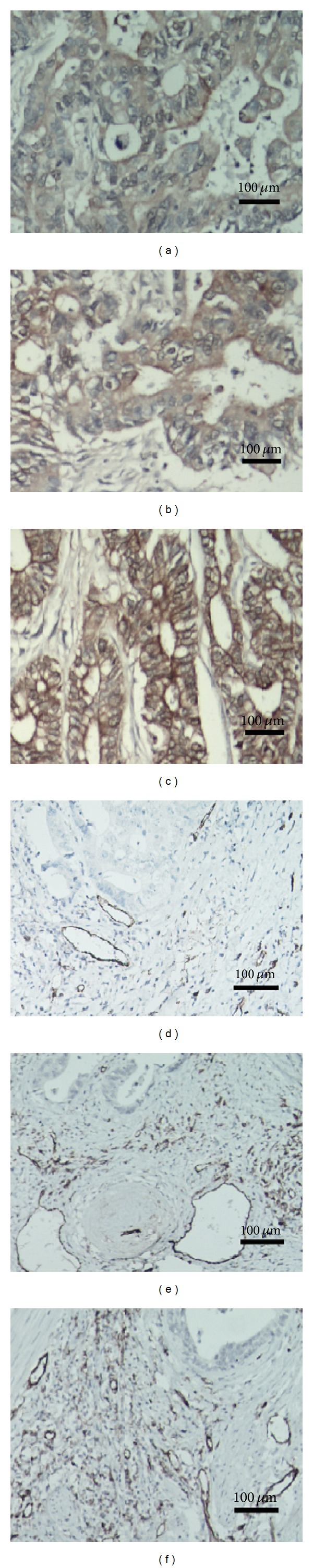
SIRT3 immunohistochemical staining in colon carcinoma tissues. SIRT3 immunohistochemical staining in colon carcinoma tissue sample from colon cancer patients. SIRT3 was mainly expressed in the cytoplasm of cancer tissues. SIRT3 is mildly expressed in the noncancerous tissue (Figure 1(a)). In colon cancer samples, the SIRT3 expression was stronger than noncancerous tissues.  Figure 1(b) shows the low expression and Figure 1(c) shows high SIRT3 expression in samples from different patient. The CD31 immunohistochemical staining in colon carcinoma tissue sample from colon cancer patients. Compared to the noncancerous tissue (Figure 1(d)), the colon cancer samples had higher CD31 expression levels.  Figure 1(e) shows the low CD31 expression and Figure 1(f) shows high CD31 expression.

Typical immunohistochemical staining of SIRT3 is shown in Figure 1. SIRT3 was mainly expressed in the cytoplasm of cancer tissues. Among all enrolled patients, high SIRT3 expression was seen in 71 patients and low SIRT3 expression was seen in 56 patients.  Table 1 shows the clinical features of these patients stratified by the SIRT3 expression patterns. We found that there was no significant correlation between the expression level of SIRT3 and patients' age (*P* = 0.415), gender (*P* = 0.052), and tumor size (*P* = 0.312); however, higher SIRT3 expression level was found to be significantly associated with tumor stages (*P* < 0.001) and lymph node metastasis (*P* < 0.001) but not transvascular metastasis (*P* < 0.001).

**Figure 2:**
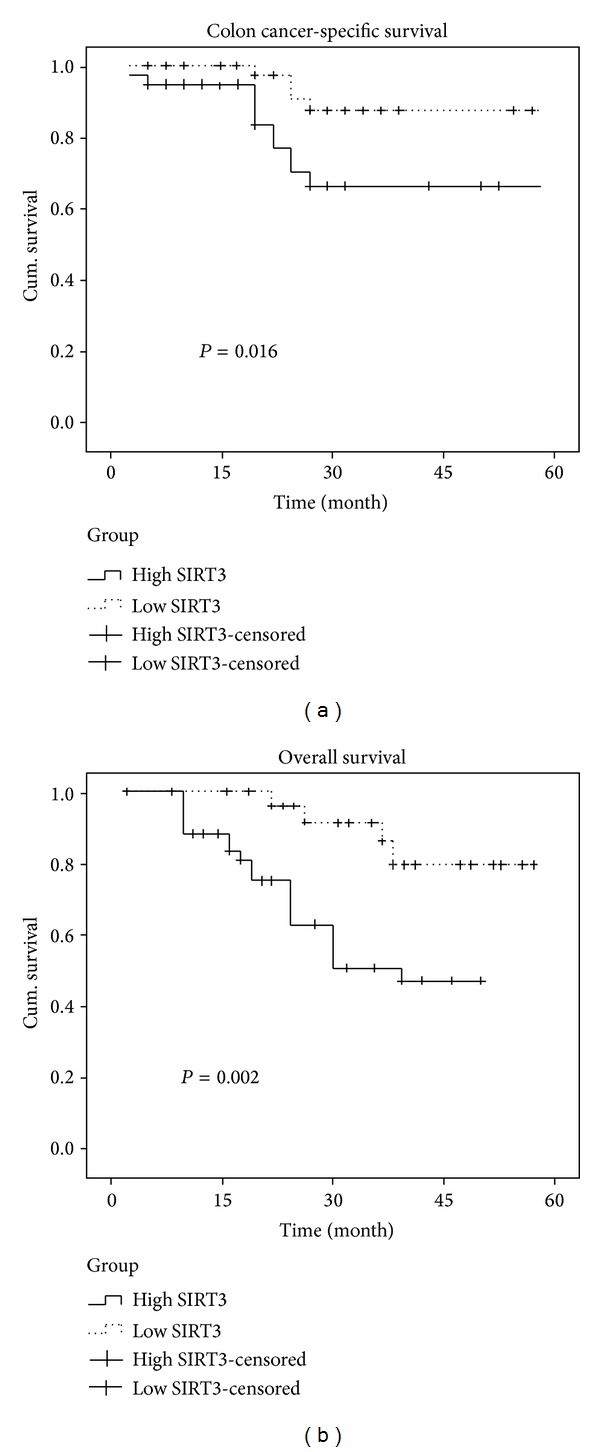
The cancer-specific survival (a) and overall survival (b) in colon cancer patients by SIRT3 expression patterns. The influence of SIRT3 expressions on patient survival was analyzed by log-rank test. Our data showed that the five-year colon cancer-specific survival was 64.6% among patients with high SIRT3 expressions and 88.6% among patients with low SIRT3 expressions (log-rank *P* = 0.016) (Figure 2(a)). Five-year overall survival was 80.2% among patients with low SIRT3 expressions and 55.9% among patients with high SIRT3 expressions (log-rank *P* = 0.002, Figure 2(b)). Censored cases were defined by nonreceipt of information about a patient's death at the time of retrieval of survival data.

For follow-up study, the median observation period was 35.3 months (ranged from 4.5 to 55.8 months). Among the 127 patients, there were 56 deaths, including 48 colon cancer-specific deaths. We assessed the influence of SIRT3 expression on patient survival by log-rank test. Our data showed that the colon cancer-specific survival rate was 64.6% among patients with high SIRT3 expressions and 88.6% among patients with low SIRT3 expressions (log-rank *P* = 0.016) (Figure 2(a)). The overall survival was 80.2% among patients with low SIRT3 expressions and 55.9% among patients with high SIRT3 expressions (log-rank *P* = 0.002, Figure 2(b).

In univariate Cox regression analysis, SIRT3 expression pattern was significantly associated with the colon cancer-specific mortality (hazard ratio (HR) 2.68; 95% confidence interval (CI), 1.48–4.28, *P* = 0.0015). In a multivariate model that is adjusted for other clinical variables, including age, sex, tumor size, tumor stage, and lymph node metastasis, SIRT3 expression remains to be associated with a significant increase in colon cancer-specific mortality (multivariate HR 2.40; 95% CI, 1.38–3.73, *P* = 0.003) and the five-year overall survival rate (multivariate HR 1.87; 95% CI, 1.12–3.26, *P* = 0.0068).

**Figure 3:**
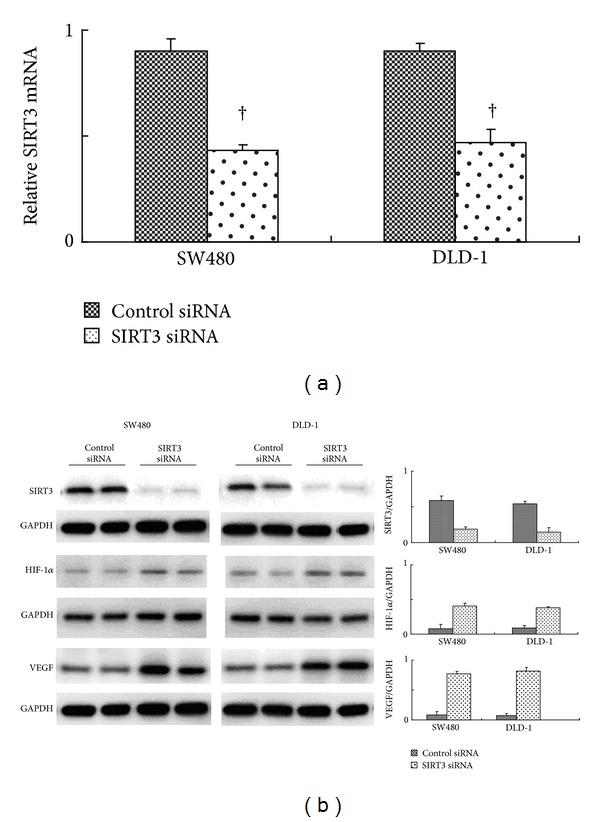
SIRT3 mRNA and protein expressions in SW480 and DLD-1 cells transfected with SIRT3 siRNA compared with cells transfected with control siRNA. (a) Real-time PCR quantitative analyses show that the SIRT3 mRNA expression levels were dramatically decreased after the SIRT3 siRNA transfection in both SW4980 and DLD-1 cell lines. (b) shows the typical western bolt bands of SIRT3, HIF1*α*, and VEGF in SW480 and DLD-1 cell lines. Quantitative analyses of relative expression ratios of SIRT3 were significantly inhibited by siRNA transfection compared to control siRNA (^†^ *P* < 0.001). Also we analyzed the HIF-1*α* expression level after SIRT3 siRNA transfection. We found that HIF1 was markedly increased in cells with low SIRT3 expression. We next analyzed the angiogenesis regulator VEGF-A expression level. The SIRT3 inhibition unregulated the VEGF-A expression levels in cultured cells.

Western blot results confirmed a significant reduction of SIRT3 protein expression in both SW480 and DLD-1 cells transfected with SIRT3 siRNA compared with cells transfected with control siRNA (Figure 3(a)). Quantitative analyses revealed that the SIRT3 expression levels were significantly inhibited in SW480 and DLD-1 cell transfected with SIRT3 siRNA (SW480: 0.78 ± 0.11 versus 0.37 ± 0.14; DLD-1: 0.83 ± 0.09 versus 0.27 ± 0.12; both: *P* < 0.001, Figure 3(b)).

**Figure 4:**
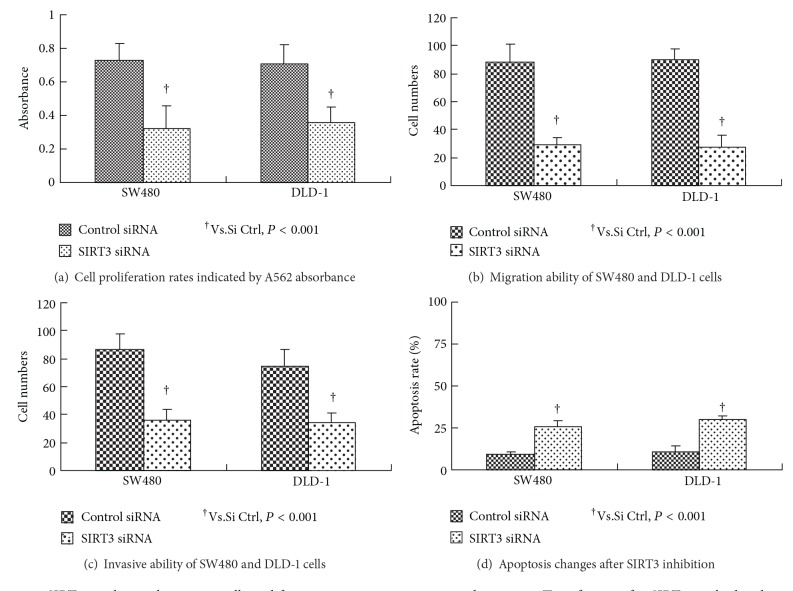
SIRT3 regulates colon cancer cells proliferation, migration, invasion, and apoptosis. Transfection of si-SIRT3 resulted in decreased cell proliferation (a), migration (b), and invasion (c) in the SW 480 and DLD-1 cells. In contrast to the above mentioned changes, the apoptosis was dramatically increased by SIRT3 inhibition in two cell lines (d).

Cell proliferation assays revealed that the growth rates were significantly inhibited in both SW480 and DLD-1 cancer cells after SIRT3 siRNA transfection (SW480: 0.75 ± 0.09 versus 0.32 ± 0.11; DLD-1 : 0.74 ± 0.13 ± 0.06; both: *P* < 0.001, Figure 4(a)). Cell migration assay showed that SIRT3 knockdown by siRNA transfection significantly decreased the cells migration ability of SW480 and DLD-1 cells (SW480: 79 ± 12 versus 34 ± 7; DLD-1 : 81 ± 12 versus 28 ± 8; both: *P* < 0.001, Figure 4(b)). Furthermore, silencing of SIRT3 gene by siRNA dramatically inhibited the invasive ability of SW480 and DLD-1 cells (SW480: 76 ± 13 versus 27 ± 11; DLD-1: 67 ± 9 versus 26 ± 10; both: *P* < 0.001, Figure 4(c)). In contrast to the above mentioned changes, the apoptosis was dramatically increased by SIRT3 inhibition in two cell lines (SW480: 26.6 ± 3.4 versus 10 ± 5.4; DLD-1: 30 ± 2.5 versus 26 ± 1.2; both: *P* < 0.001, Figure 4(d)).

**Figure 5:**
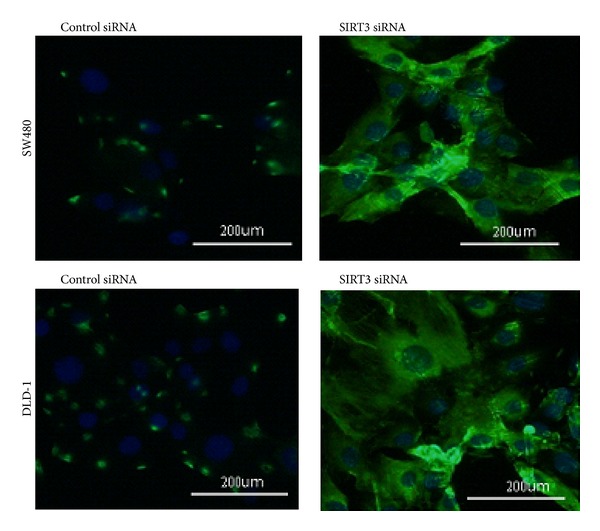
ROS formation in cells with or without SIRT3 inhibition; CM-H2DCFDA staining shows the ROS formation (green staining area) in cells counterstained with DAPI (blue staining area). SIRT3 inhibition dramatically increased the ROS formation in SW480 and DLD-1 cell lines.

We next used the CM-H2DCFDA staining to detect the ROS formation in cells. As shown in Figure 5, the SIRT3 inhibition dramatically increased the ROS formation in SW480 and DLD-1 cell lines compared to control si-RNA transfection.

## 4. Discussion

The SIRT family members (SIRT 1–7) are closely linked to carcinogenesis; carcinogenic roles of SIRT2 and SIRT7 have been documented in glioma and thyroid cancer, respectively [14, 15, 22]. SIRT3 is the only member that has been associated with longevity in human and has been identified as a cell survival factor protecting cells from genotoxic stress through utilizing the mitochondrial NAD pool in tumor cells [23]. Association of SIRT3 with breast, oral, and bladder carcinoma has been previously described [14, 15, 22]. Colon cancer is one of most prevalent malignant tumors worldwide causing high morbidity and mortality [24]. Pertinence of this study is to address the role of SIRT3 in colon cancer and to explore its potential application in colon cancer treatment.

This is a retrospective study conducted among 127 Chinese colon cancer patients. Our study indicates that high SIRT3 expression in the cytoplasm significantly correlates with high tumor grades, positive lymph node status, and poor prognosis. SIRT3 is universally expressed and prevalent in metabolically active tissues [25]. Studies have shown that it is located in both the nucleus and the mitochondria. Nuclear SIRT3 enters into mitochondria upon cellular stress induced by its own overexpression [23, 25, 26]. Cell stress initiates accumulative damage of mitochondria, resulting in decreased NAD/NADH ratio and the deacetylase activity, concomitant reduction of SIRT3 activity, and ultimately growth arrest [15]. On the contrary, increased SIRT3 expression facilitates cell growth via targeting Ku70 and promoting Ku70-Bax interaction [22, 23]. In addition, increased SIRT3 level abrogates P53 activity, a tumor suppressor protein and mitochondrial protein [14].

Discrepancy of the role of SIRT3, however, has been described as a tumor suppressor in mouse. For instance, it has been shown that the SIRT3^−/−^ mice develop ER/PR-positive mammary tumors. Expression of a single oncogene (Myc or Ras) in SIRT3^−/−^ mouse embryonic fibroblasts (MEFs) resulted in* in vitro* transformation and immortalization of MEFs [27]. Nevertheless, overexpression of SIRT3 was correlated to the incidences of oral squamous cell carcinoma (OSCC) and downregulation of SIRT3 improved the sensitivity of OSCC to radiation and chemotherapy at the same time [14]. This may be due to the complex function of SIRT3 that varies depending on studied tissues or origin, as examined in the function of SIRT1 by Alhazzazi et al. [14, 28].

In our study, western blot results demonstrate significant reduced SIRT3 protein expression that occurred following transfection of SW480 and DLD-1 with SIRT3 siRNA compared with cells transfected with the nonspecific siRNA control. This data corresponds to decreased proliferation, migration, and invasion of SW480 and DLD-1 cells presented* in vitro* after treatment with siRNA SIRT3. Promise of siRNA-based therapy has been shown in many diseases including cancer. This type of therapy may potentially reduce resistance to chemotherapy when combined with standard chemotherapy in certain cancers [12, 29–31]. Although molecular mechanisms of SIRT3 in carcinogenesis are not completely clear, this finding encourages further study on SIRT3-based treatment in colon cancer.

We did the* in vitro* study to analyze the HIF1*α* expression levels after SIRT3 inhibition. Consistent with the findings of Yang et al. [32], we found that knockdown of SIRT3 significantly increased the expression of HIF-1*α* in SW480 and DLD-1 cells. Also we detected the VEGF-A expression level* in vitro*, finding that the SIRT3 KO will increase the VEGF expression.

Occurrence of colon cancer has been shown as a consequence of interaction between genetic predisposition and environmental factors, including epidemic obesity and sedentary lifestyle [33]. Combined effects from six individual factors, that is, dietary intake, alcohol consumption, smoking, physical activity, body mass index, and sleep patterns, have been associated with incidence of colon cancer in non-Western populations, whereas the Western dietary pattern has been found in association with CRC in non-Asian populations [34–37]. Stratification of risk factors was not performed in statistical analysis of this study due to sample size and the complexity of etiology in colon cancer, which is outside the scope of this paper. Even though development of colon cancer originates from stem cells and “mucosa at risk” through various pathways following different responses to DNA damage [33, 38–41], common outcomes are presented at the cellular and molecular level and manifested by several caner hallmarks and molecular characteristics [42–44].

The mechanisms of SIRT3 promoting tumor cell survival may be due to both decreased apoptosis and increased cell proliferation, both of which are hallmarks of colon cancer carcinogenesis. Overall, our data suggests that enhanced SIRT3 expression is associated with colon carcinoma growth and spread among a cohort of Chinese colon cancer patients. Patients with low SIRT3 expression had improved five-year colon cancer-specific survival and overall survival compared to patients with high SIRT3 expression determined by the IRS score. The mechanisms of SIRT3 promoting tumor cell survival may be due to increased cell proliferation, migration, and invasion as suggested by our* in vitro* study. Further study of SIRT3 in a larger population as well as in different ethnical groups is warranted in order to better understand prognostic and therapeutic values of SIRT3 as a biomarker in colon cancer. Treatment targeting the SIRT3 protein may be alternative or adjunctive to current chemotherapy for colon cancer patients.

## Figures and Tables

**Table 1:** The expression level of SIRT3 and clinical features.

Variable	Patients	SIRT3 expression		*P* value
	Total = 127	High = 71	Low = 56	
Gender				
Male	50	23	27	
Female	77	48	29	0.052
Age				
≥60	66	38	28	
<60	61	33	28	0.415
Tumor size				
≥5 cm	57	30	27	
<5 cm	70	41	29	0.312
Tumor stages				
I-II	42	18	24	**<0.001**
III-IV	85	53	32	
Lymph node metastasis				
Presence	63	46	17	
Absence	64	25	39	**<0.001**
Transvascular metastasis				
Presence	45	25	20	**0.550**
Absence	82	46	36	
